# Regulation of TEAD Transcription Factors in Cancer Biology

**DOI:** 10.3390/cells8060600

**Published:** 2019-06-17

**Authors:** Hyunbin D. Huh, Dong Hyeon Kim, Han-Sol Jeong, Hyun Woo Park

**Affiliations:** 1 Department of Biochemistry, College of Life Science and Biotechnology, Yonsei University, Seoul 03722, Korea; huhhb621@yonsei.ac.kr (H.D.H.); dhk1107@yonsei.ac.kr (D.H.K.); 2 Division of Applied Medicine, School of Korean Medicine, Pusan National University, Yangsan, Gyeongnam 50612, Korea

**Keywords:** TEAD, Hippo pathway, cancer, stem cell

## Abstract

Transcriptional enhanced associate domain (TEAD) transcription factors play important roles during development, cell proliferation, regeneration, and tissue homeostasis. TEAD integrates with and coordinates various signal transduction pathways including Hippo, Wnt, transforming growth factor beta (TGFβ), and epidermal growth factor receptor (EGFR) pathways. TEAD deregulation affects well-established cancer genes such as KRAS, BRAF, LKB1, NF2, and MYC, and its transcriptional output plays an important role in tumor progression, metastasis, cancer metabolism, immunity, and drug resistance. To date, TEADs have been recognized to be key transcription factors of the Hippo pathway. Therefore, most studies are focused on the Hippo kinases and YAP/TAZ, whereas the Hippo-dependent and Hippo-independent regulators and regulations governing TEAD only emerged recently. Deregulation of the TEAD transcriptional output plays important roles in tumor progression and serves as a prognostic biomarker due to high correlation with clinicopathological parameters in human malignancies. In addition, discovering the molecular mechanisms of TEAD, such as post-translational modifications and nucleocytoplasmic shuttling, represents an important means of modulating TEAD transcriptional activity. Collectively, this review highlights the role of TEAD in multistep-tumorigenesis by interacting with upstream oncogenic signaling pathways and controlling downstream target genes, which provides unprecedented insight and rationale into developing TEAD-targeted anticancer therapeutics.

## 1. Introduction

The TEAD family of transcription factors are the final nuclear effectors of the Hippo pathway, which regulate cell growth, proliferation, and tissue homeostasis via their transcriptional target genes. Since their initial discovery three decades ago, TEADs have been best studied in the context of the Hippo-YAP/TAZ signaling pathway and tumorigenesis. To date, studies on TEAD activity are limited to serving as the functional readout of the Hippo-YAP/TAZ pathway. However, recent evidence suggests that nucleocytoplasmic shuttling, post-translational modifications, and crosstalk between oncogenic signaling pathways are important determinants of TEAD activity both in vitro and in vivo. Importantly, since the Hippo pathway components are hardly druggable, TEADs have emerged as critical drug candidates to treat human diseases including cancer, cardiovascular diseases, and neurodegenerative disorders. The current review underscores and reinterprets the oncogenic role of TEADs in tumorigenesis in past reports from a TEAD point of view in order to provide unprecedented insight and rationale into developing TEAD-targeted anticancer therapeutics.

## 2. The TEAD Family of Transcription Factors

**Figure 1:**
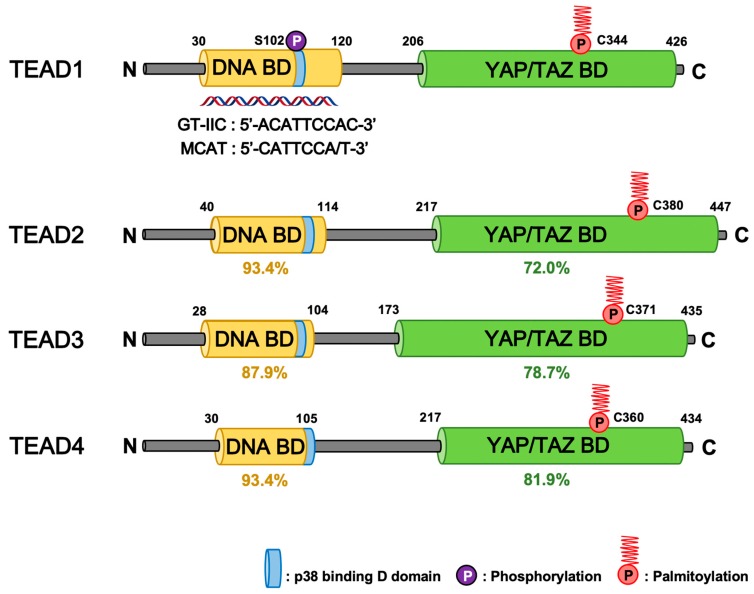
Domain architecture of human TEADs. The N-terminal DNA binding domain (DNA-BD) and C-terminal YAP/TAZ binding domain (YAP/TAZ-BD) of TEAD1-4 harbor high similarity across four different paralogs. The percent (%) represents the identity for each domain of TEADs compared to that of TEAD1 [50]. TEAD post-translation modifications include palmitoylation and PKA-, PKC-mediated phosphorylation that occur in the YAP/TAZ-BD and DNA-BD, respectively. Palmitoylation is required for proper TEAD functions. TEAD cytoplasmic translocation occurs through protein-protein interaction with p38 MAPK that binds the p38-binding motif within the DNA-BD of all TEADs.

**Figure 2:**
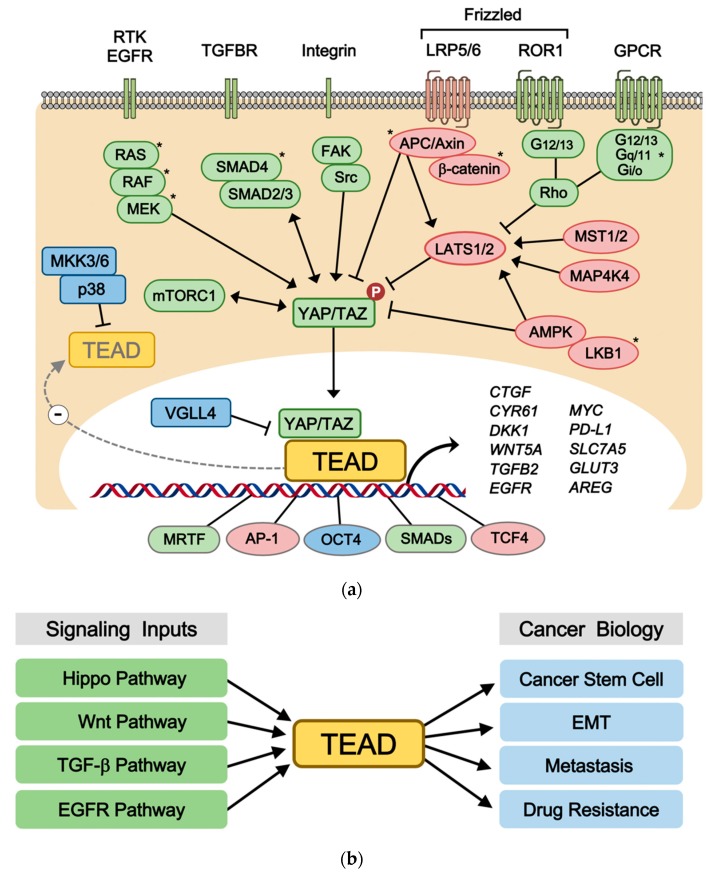
The regulatory mechanisms of TEAD in cancer biology. (**a**) Upstream signaling and downstream transcriptional outputs of TEAD. Various oncogenic signal transduction pathways, such as EGFR signaling, TGFβ signaling, Wnt signaling, GPCR signaling, and cancer genes (*), such as KRAS, BRAF, LKB1, APC, GNAQ/11 regulate TEAD activity through multiple signaling mechanisms. The TEAD transcriptional outputs have critical functions in tumorigenesis, stem cell maintenance, cancer immunology, metabolism as well as formation of signaling feedback loops. (**b**) Role of TEAD in multiple stages of tumorigenesis. TEAD activation via various oncogenic pathways play critical roles in cancer biology including EMT, metastasis, drug resistance, and cancer stem cells.

All four TEADs are transcription factors that are evolutionarily conserved and broadly expressed in most tissues of the human body [1,2]. Each family member has multiple names TEAD1 (TEF-1/NTEF), TEAD2 (TEF-4/ETF), TEAD3 (TEF-5/ETFR-1), and TEAD4 (TEF-3/ETFR-2/FR-19). Despite the high homology and expression pattern shared between TEAD1-4, animal model experiments indicate that each TEAD has tissue-specific roles, such as cardiogenesis, neural development, and trophectoderm lineage determination, during embryonic development [3,4,5,6,7,8]. One of their major roles in cell biology is to regulate cell proliferation and contact inhibition [9,10]. TEADs also share highly similar domain architectures (Figure 1). TEAD N-terminus share a highly conserved 68-amino acid TEA/ATTS DNA-binding domain, which binds to the MCAT element (5′-CATTCCA/T-3′) originally defined as the GT-IIC motif (5′-ACATTCCAC-3′) of the simian virus 40 (SV40) enhancer [11,12,13]. Based on these sequences, a synthetic TEAD luciferase reporter 8xGTIIC-luciferase plasmid, which contains eight GT-IIC motifs, is being widely used to measure YAP/TAZ and TEAD activity [14]. Unlike several other transcription factors, TEAD is known to be mostly bound to the DNA since the majority of TEADs are found in the chromatin fraction [15]. However, they hardly exhibit any transcriptional activity by themselves [16]. Hence, TEAD activity mainly relies on the C-terminus, in which all TEADs share their transactivation domain in order to recruit transcriptional coactivators YAP/TAZ [17,18,19,20], corepressors VGLL1-4 [21,22,23,24,25,26], chromatin remodeling factors NuRD [27], and the Mediator [28]. Although TEAD has been recognized as the final effector of the Hippo-YAP/TAZ pathway, TEAD also interacts with other signaling transduction pathway transcription factors including TCF, SMAD, OCT4, AP-1, and MRTF [29,30,31,32,33] (Figure 2a). TEAD-driven transcriptional targets include well-established genes that are involved in cell growth, proliferation, and tissue homeostasis. In addition to classical TEAD target genes such as *CTGF* and *CYR61* [18], recent studies also identify W*NT5A/B* [34], *DKK1* [34,35], *TGFB2* [36], *BMP4* [37], *AREG* [38], *EGFR* [39], *PD-L1* [40,41,42,43], *MYC* [44,45], *LATS2* [46], amino acid transporters *SLC38A1/SLC7A5* [47,48], and glucose transporter *GLUT3* [49] as direct TEAD target genes (Figure 2a). These signaling inputs, protein-protein interactions, and target genes further expand the roles of TEAD to directly control Wnt, TGFβ, RTK, mTOR, and Hippo signaling in the context of tumorigenesis, cancer immunity, stem cell pluripotency, metabolism, and development.

## 3. Signaling Inputs and Transcriptional Outputs of TEAD

### 3.1. Hippo Pathway

Since TEADs exhibit minimal transcriptional activity by themselves, they require coactivators to induce target gene expression [16]. The most well-established cofactors that activate TEAD-mediated transcription are YAP and its paralog TAZ, which are transcriptional coactivators of the Hippo pathway that play major roles in organ size control, cell proliferation, tumorigenesis, and stem cell self-renewal [51,52,53,54] (Figure 2a). The N-terminus of YAP/TAZ interact with the C-terminal transactivation domain of TEAD to form a YAP/TAZ-TEAD complex that constitutes the nuclear transcriptional module of the Hippo pathway [55]. On the other hand, the cytosolic kinase modules of the Hippo pathway, which consists of MST1/2, MAP4K4, and LATS1/2, phosphorylate YAP/TAZ at multiple sites. This promotes YAP/TAZ cytoplasmic retention, ubiquitination, and protein degradation [56]. Cytoplasmic YAP/TAZ are degraded by both the ubiquitin-proteasome system and autophagy [57,58,59,60], which renders TEAD transcriptionally inactive.

To date, numerous studies and ChIP-seq analyses highlight YAP/TAZ to be the major TEAD coactivators. In MDA-MB-231 breast cancer cells that harbor genetic inactivation of the Hippo pathway (*NF2* null), approximately 80% of TEAD4-bound promoters and enhancer regions were co-occupied with YAP/TAZ, while the TEAD consensus sequence was present in 75% of DNA-bound YAP/TAZ peaks [32]. In MCF10A mammary gland epithelial cells, YAP and TEAD1 co-occupied 80% of the promoters [18]. Furthermore, in glioblastoma cells, 86% of all YAP peak regions contained at least one TEAD binding site [61]. Although YAP/TAZ can interact with different transcription factors such as RUNX, p73, KLF4, TBX5, SMAD, and others, TEADs are the predominant factors that facilitate YAP/TAZ recruitment to the chromatin. In mouse studies, dominant-negative TEAD2 was found to be sufficient in suppressing YAP overexpression-, or NF2 inactivating mutation-induced hepatomegaly and tumorigenesis, which indicates that TEAD mostly attributes to YAP-induced tumorigenesis [62]. Although oncogenic driver mutations have not been reported in TEADs, numerous studies demonstrate their pro-tumorigenic roles due to their crosstalk with other cancer genes, which is discussed in later sections. 

Furthermore, studies have shown that TEAD interaction is required for YAP/TAZ nuclear retention. Osmotic stress-activated p38 MAPK induces TEAD cytoplasmic translocation independent of the Hippo-YAP/TAZ pathway. In this context, YAP/TAZ fails to accumulate in the nucleus regardless of its phosphorylation status [63]. In TEAD knockout cells, YAP/TAZ remained in the cytoplasm even after treating YAP/TAZ activating stimuli such as LPA and serum [63], and, similarly, TAZ mutants defective in terms of TEAD interaction showed impaired nuclear accumulation [64]. In addition, forced expression of wild-type TEAD2, but not the YAP/TAZ binding deficient-TEAD2 mutant, induced nuclear localization of YAP/TAZ, which was required for tumorigenesis [65]. These results suggest a possible mechanism by which the TEAD interaction is required for YAP/TAZ accumulation and activation in the nucleus. 

Moreover, the TEAD transcriptional output also regulates Hippo-YAP/TAZ signaling via target gene expression. TEAD target genes include important membrane transporters and secreted ligands that act through autocrine or paracrine signaling. The *AREG*, *WNT5A/B*, and *TGFB2* ligands activate YAP/TAZ via EGFR [66,67], Frizzled/ROR [34,68,69], and TGFBR [70,71,72], respectively, while *SLC38A1/SLC7A5* and *GLUT3* activate YAP/TAZ via mTORC1 [58] and glycolytic enzymes [49,73] respectively. Therefore, these TEAD-dependent positive feedback loops function as a nexus that coordinates important biological pathways and deregulation may lead to human malignancies. TEADs also induce *LATS2*, *NF2*, and *AMOTL2*, which are major components of the Hippo pathway, to form a negative feedback loop [46,74]. Thus, these TEAD-mediated feedback loops provide an efficient mechanism by which the robustness and homeostasis of Hippo-YAP/TAZ regulation is established.

### 3.2. Wnt Pathway

The canonical Wnt/β-catenin pathway and alternative Wnt pathway are one of the best characterized upstream signal transduction pathways that regulate TEAD. Crosstalk between the Wnt and Hippo pathways converge on TEAD via destruction complex-dependent and -independent mechanisms, which have been studied in the context of tumorigenesis, stem cell biology, and development. In these contexts, TEAD activity is indispensable for Wnt-induced biological responses (Figure 2a).

Canonical Wnt/β-catenin pathway regulates TEAD and YAP/TAZ via both Hippo-dependent and -independent mechanisms. Using immunoprecipitation assay, YAP/TAZ were shown to directly interact with the β-catenin destruction complex consisting of Axin1, APC, and GSK3β [75,76]. Within the destruction complex, YAP bridges β-TrCP E3 ligase to degrade β-catenin. Another study shows that APC binds and activates the Hippo pathway components, LATS1 and Sav1, which, in turn, inhibits YAP/TAZ [77]. In both cases, Wnt stimulation or loss-of-function mutations in APC trigger YAP/TAZ nuclear translocation and TEAD-mediated transcription. Furthermore, YAP/TAZ mediates >50% of the Wnt target genes induced by APC deletion in transformed mammary epithelial cells [75]. TEAD4 also directly interacts with TCF4, and the TEAD4-TCF4 complex directly links Wnt/β-catenin and Hippo/YAP signaling at the transcription factor level [29]. Thus, TEAD transcriptional output underlies the pathogenesis of APC mutation-induced colorectal tumorigenesis and Wnt-induced crypt regeneration.

The alternative Wnt pathway, which is independent of β-catenin and the destruction complex, also plays an important role in tumorigenesis, differentiation, development, and Wnt/β-catenin signaling inhibition. The alternative Wnt ligand Wnt5A/B is a potent TEAD activator that signal via the Frizzled/ROR1-Gα_12/13_-Rho GTPases-Hippo-YAP/TAZ pathway in the context of cancer progression and mesenchymal stem cell differentiation [34]. Subsequent studies further demonstrate YAP/TAZ and TEAD activation via the alternative Wnt pathway in the context of breast cancer progression, chemotherapy resistance, stem cell maintenance, and macrophage polarization [68,69,78,79]. 

Moreover, the TEAD transcriptional output regulates the Wnt pathway. Major TEAD target genes that inhibit Wnt/β-catenin pathway are *DKK1* and *WNT5A/B*. These are well-established secreted inhibitors of the canonical Wnt/β-catenin pathway [34,35]. Wnt3a stimulation induces *WNT5A/B* gene expression via TEAD, which suppresses Wnt/β-catenin-induced mesenchymal stem cell differentiation [34]. In addition, YAP represses *WNT3* gene expression to inhibit the Wnt/β-catenin pathway possibly via TEAD [80]. Although YAP has often been shown to inhibit Wnt-induced biological responses in cancer and stem cells [35,81,82,83], the precise involvement of the TEAD transcriptional output requires further investigation. Collectively, TEADs are activated via the upstream Wnt pathway, while the TEAD transcriptional output concomitantly inhibits Wnt signaling, thus forming a negative feedback loop.

### 3.3. TGFβ Pathway

The TGFβ pathway regulates multiple biological processes including embryonic development, stem cell differentiation, immune regulation, wound healing, and inflammation. The crosstalk between the TGFβ and Hippo pathway centers on Smad and TEAD transcription factors, respectively. TGFβ stimulation triggers TEAD-mediated biological responses in the context of cell fate determination, tumorigenesis, and fibrosis, which are either dependent or independent of Smads (Figure 2a). TGFβ increases the expression level and activity of TEAD, and vice versa, TEAD can also directly trigger TGFβ signaling. TGFβ induces TAZ expression via a Smad3-independent, p38-mediated, and MRTF-mediated mechanism [84]. TGFβ also induces TEAD2 expression during epithelial-to-mesenchymal transition (EMT) [65]. Thus, TGFβ-induced TEAD target gene expression promotes EMT in mammary gland epithelial cells and malignant tumor phenotypes. Notably, the TGFBII ligand itself is a direct target gene of TEAD that evokes a positive feedback regulation [36,85].

Upon TGFβ stimulation, Smad2 and Smad3 form complexes with Smad4 and accumulate in the nucleus. It is important to note that TGFβ-induced Smad nuclear translocation is dependent on YAP/TAZ. For example, lung and breast cancers lacking RASSF1A display hyperactive TGFβ signaling and tumor invasion via the YAP-dependent nuclear localization of Smad2 [70]. In human embryonic stem cells, TAZ is also required for TGFβ-induced nuclear translocation of the Smad2/3-4 complex, and TAZ-dependent Smad2/3 nuclear translocation is required to maintain self-renewal markers [71]. YAP/TAZ also regulates the localization of the Smad complex in response to cell density-mediated formation of polarity complexes [86]. However, the direct involvement of TEAD in YAP/TAZ-dependent Smad localization requires further investigations. 

It is still unknown how TGFβ signaling switches from enforcing pluripotency to promoting mesendodermal differentiation during development. Smad2/3 forms an enhancer complex with TEADs and OCT4, which suppress the gene expression of differentiation markers and modulates the levels of core pluripotency genes. This maintains the pluripotency of embryonic stem cells [30]. An independent study showed that TEAD binds the negative elongation factor and blocks Wnt3a/β-catenin and Activin/Smad2/3-induced mesendodermal differentiation [87]. TEAD also mediates TGFβ-induced tumorigenesis. The majority of malignant mesotheliomas harbor genetic inactivation of the Hippo pathway components, which collaborate with the TGFβ pathway by forming the YAP-TEAD4-Smad3-p300 complex on the CTGF promoter to induce gene expression and tumor growth [88]. Furthermore, a multitude of genes harbor both Smad-binding and TEAD-binding elements in their promoters. In breast cancer cells, TGFβ-YAP/TAZ-TEAD signaling is crucial in driving late-stage metastatic phenotypes via Smad2/3-induced *NERG1* and *UCA1* transcription [72]. Accumulating evidence highlights the importance of the TEAD transcriptional output acting as a bona fide effector of the TGFβ pathway during development and tumorigenesis. 

## 4. Molecular Mechanisms Controlling TEAD Activity

### 4.1. Regulation of TEAD via Subcellular Localization

Transcription factors have been shown to form distinct protein complexes both on and off the chromatin [15]. Important signaling pathways function by ultimately regulating the activity and subcellular localization of transcription factors. For example, the final effectors of the TGFβ, Wnt, NFκB, and Hippo signaling pathways are Smads, TCF/LEF, p65, and TEAD transcription factors, respectively, and their chromatin association is tightly controlled via complex upstream signals. Unlike many other transcription factors, a majority of TEAD proteins reside on the chromatin [15], which are known to be passively regulated and dependent on cofactors such as YAP/TAZ and VGLL4. However, mechanistic insight that governs dynamic TEAD subcellular localization was recently elucidated. 

There is a wide range of cellular stress, such as energy starvation, oxidative stress, cytotoxic agent, which induce Hippo kinase activation, YAP/TAZ phosphorylation, cytoplasmic translocation, protein degradation, and thus inhibition of TEAD transcriptional outputs. However, these stimuli did not alter TEAD subcellular localization or expression levels. Interestingly, certain environmental stresses, such as osmotic stress, high cell density, and cell suspension, promoted TEAD cytoplasmic translocation [89]. Although controversial, the cytoplasmic localization of TEAD4 has been observed in the inner cell mass of mouse embryonic stem cells [90,91]. Cytoplasmic TEADs were also detected in lung, spleen, and kidney tissues, but not in renal cell carcinoma tissues [63]. A splicing isoform of TEAD4, which lacks the N-terminal DNA binding domain, is found in the cytoplasm acting as a dominant negative isoform that inhibits YAP activity [92]. Because TEADs are the major effectors that dictate the transcriptional output of the Hippo-YAP/TAZ pathway, physiologic and pathologic conditions affecting TEAD localization significantly impact the functional output of the Hippo pathway.

The molecular mechanism of stress-induced cytoplasmic translocation of TEAD involves the p38 MAPK pathway, which is independent of Hippo pathway components [89]. Therefore, the crosstalk between p38 MAPK and the Hippo pathway impinges on TEAD (Figure 2a). The p38-binding motif is located near the N-terminus nuclear localization signal (NLS) of TEADs through which TEAD-p38 forms a complex via direct protein-protein interaction (Figure 1). Although p38 does not phosphorylate TEAD directly, its kinase activity is required for TEAD-p38 complex formation and subsequent cytoplasmic translocation. Cellular responses to environmental stresses are mediated by distinct gene expression during the acute and adaptation phases [93]. During the acute phase of osmotic stress, YAP-TEAD is activated via NLK-mediated YAP S128 phosphorylation [94,95]. However, during the adaptation phase to stress, p38 binds and inhibits TEAD by inducing its cytoplasmic translocation, which indicates that the TEAD transcriptional output is required for cell survival upon the acute stress response, but is indispensable during the adaptation phase.

More importantly, the nuclear absence of TEAD impairs YAP/TAZ nuclear accumulation [63,64,65]. For example, in TEAD 1/2/4 KO cells, LPA-stimulated YAP/TAZ fails to accumulate in the nucleus even after it is completely dephosphorylated [63]. Therefore, stimuli that evoke TEAD cytoplasmic translocation overrides YAP/TAZ activating signals. The mechanism of stress- and p38-induced TEAD cytoplasmic retention is intact in various cancer cells. Notably, YAP-driven cancer cells, such as the GNAQ/11 mutant uveal melanoma cells and Hippo mutant mesothelioma cells, were specifically sensitive to TEAD cytoplasmic translocation when compared to YAP-independent cancer cells [63]. These findings suggest that signal transductions and/or chemical compounds that modulate TEAD subcellular localization potentially predominate the biological function of the Hippo-YAP/TAZ pathway.

### 4.2. Regulation of TEAD via Post-Translational Modifications

The post-translational modifications in TEAD have recently gained significant interest after its important roles in human pathophysiology came to light. To date, TEAD phosphorylation and palmitoylation have been shown to regulate its function. TEAD is phosphorylated by protein kinase A (PKA) and protein kinase C (PKC), which have been shown to inhibit TEAD by disrupting its DNA-binding [96,97] (Figure 1). However, the precise mechanism and context of TEAD phosphorylation requires further investigation. Recently, TEAD palmitoylation emerged as an important post-translational regulation mechanism [98,99,100]. Protein palmitoylation is important for protein trafficking and membrane localization [101]. S-palmitoylation of TEAD occurs by attaching a fatty acid (palmitate) to conserved cysteine residues in the TEAD C-terminus, which are within the YAP-binding domain (YBD) that share > 70% sequence identity among TEAD1-4 (Figure 1). The palmitoyl group is buried deep inside a hydrophobic pocket of TEAD as revealed by structural studies. All four TEAD paralogs are found palmitoylated in mammalian cells via an autopalmitoylation process that could be removed via a certain depalmitoylating enzyme such as APT2 [99,102]. Although the molecular mechanisms of YAP/TAZ and TEAD regulation via TEAD-YBD palmitoylation have been proposed, the functional role of YBD palmitoylation still remains unclear. Noland et al. and Mesrouze et al. showed that TEAD palmitoylation did not alter protein localization and YAP/TAZ binding. However, it was required for proper TEAD folding and protein stability [99,100]. Impaired TEAD2 palmitoylation decreased TEAD protein stability and resulted in the loss of protein abundance. On the other hand, Chan et al. demonstrated that TEAD palmitoylation did not affect its protein stability. However, it was required for the YAP/TAZ interaction. Palmitoylation-deficient TEAD mutants were not able to bind YAP/TAZ. Therefore, TEAD transcriptional activity was impaired, which indicates that TEAD palmitoylation plays important roles in regulating its binding to the transcriptional coactivators. Palmitoylation-deficient mutants have been reported to be properly folded and could still bind VGLL4, which suggests that the loss of YAP/TAZ binding is not due to TEAD misfolding [98]. The palmitoylation-deficient TEAD1 mutant also impaired TAZ-mediated muscle differentiation and YAP-mediated tissue overgrowth in Drosophila. 

Thus, the precise role of TEAD palmitoylation requires in-depth investigation since the palmitate-binding hydrophobic pocket located in the YBD is likely to be an important site for therapeutic intervention. The first indication that the hydrophobic pocket could be a therapeutic target was obtained from a high-throughput screening that attempt to identify ligands that stabilized the TEAD-YBD. The results indicated that NSAIDs, such as flufenamic acid (FA) and niflumic acid (NA), were small-molecular inhibitors that bound the central TEAD hydrophobic pocket at its palmitoylation site [103]. Although the affinity of these drugs to TEAD requires optimization, FA- and NA-treatments decreased TEAD transcriptional activity and TEAD-induced cell migration and proliferation without disrupting TEAD-YAP interaction. Whether FA and NA compete against TEAD palmitoylation requires further investigation.

## 5. Roles of TEAD in Cancer Biology

### 5.1. TEAD Expression in Human Cancers

Numerous studies suggest the importance of TEAD in the development of human cancers. TEAD overexpression and hyperactivity has been implicated in multiple stages of cancer progression (Figure 2b). Although TEAD may be downregulated in some breast cancers and renal or bladder tumors [104], high TEAD expression levels have been correlated with poor clinical outcome, which serves as a prognostic marker in various solid tumors, such as prostate cancers [105], colorectal cancers [106,107], gastric cancers [108,109], breast cancers [110,111], germ cell tumors [112,113], head and neck squamous cell carcinomas [114], renal cell carcinomas [115], and medulloblastomas [116]. On the other hand, a loss-of-function mutation in TEAD1 (Y421H) was shown to cause Sveinsson’s chorioretinal atrophy, which is a genetic disorder that results in degeneration by disrupting TEAD1-YAP interaction [117,118]. In accordance with these studies, meta-analysis studies revealed that both total and nuclear YAP and TAZ expression are intimately associated with adverse overall survival (OS) and disease-free survival (DFS) in numerous cancers, which suggests the prognostic role of TEADs and YAP/TAZ expression in patients with various malignancies [119,120]. Notably, TEAD was shown to be a critical drug target in YAP-driven tumorigenesis. In hepatocellular carcinoma (HCC), dominant-negative TEAD reversed YAP-induced hepatomegaly and tumorigenesis in vivo. Moreover, VGLL4-mimicking peptide (which binds the YBD) and verteporfin (a small YAP-binding chemical) were demonstrated to harbor therapeutic effects against YAP-induced tumorigenesis by interrupting TEAD-YAP interaction [62,121,122].

### 5.2. Role of TEAD in EMT

TEADs have emerged as important drivers of cancer development, tumor growth, EMT, metastasis, and drug resistance (Figure 2b). EMT is a natural developmental process that is phenocopied by cancer cells of epithelial origin. This process is crucial for cancer cells since it promotes cell migration, invasion, and anoikis resistance. Therefore, EMT emerged as a critical regulator of the cancer stem cell phenotype and a prerequisite for metastasis [123]. TEADs are critical mediators of EMT and metastasis during cancer progression. Numerous studies indicate that the TEAD transcriptional output induced by YAP/TAZ activation drives cell transformation by inducing EMT [17,18,124,125,126]. In these studies, TEAD activation disrupted cell-cell junctions, promoted mesenchymal gene expression, and increased cell migration and invasion. Aberrant TEAD activity was shown to promote mammary carcinoma and melanoma metastasis in a manner that was highly dependent on the YAP-interaction domain, which suggests that the interaction between TEAD and YAP is essential for EMT and metastasis [126]. TEAD also contributes to EMT by upregulating Slug and ZEB1 [127,128], which are major EMT transcription factors that promote cell migration and invasion by inhibiting epithelial markers and upregulating mesenchymal markers. TEAD triggers transcriptional induction of ZEB1 that drives metastatic squamous cell carcinoma. ZEB1 and TEAD also forms a complex to promote cancer stem cell traits and predict poor survival, therapy resistance, and increased metastatic risk in breast cancer [129]. In line with ZEB1, TEAD directly transcribes ZEB2 and represses DNp63 to regulate cell fate and lineage conversion in lung cancer progression [130]. Moreover, TEAD mediated non-small-cell lung carcinoma (NSCLC) aggressiveness by inducing Slug transcription and EMT [128]. Collectively, accumulating evidence indicates that TEAD functions as a critical EMT transcription factor in tumorigenesis. It will be interesting to answer key questions regarding the role of TEAD in EMT during development and MET (mesenchymal-to-epithelial transition), which occurs during metastatic colonization.

### 5.3. Role of TEAD in Metastasis

The majority of cancer-associated deaths occur due to metastasis, which is the dissemination of cancer cells from the primary tumor site to secondary organs. In order to metastasize, cancer cells must avoid anoikis (detachment-induced apoptosis) and must enter and exit from the blood vessels (intravasation and extravasation, respectively), complete metastatic colonization, and acquire drug resistance. These are all hallmarks of cancer stem cells. Upon interaction with platelets, metastatic cancer cells induce TEAD activation via the RhoA-MYPT1-PP1-YAP pathway. The platelet-activated TEAD transcriptional program in detached cancer cells induced anoikis-resistance and promoted cell survival and metastasis [131]. Similarly, YAP promotes anoikis-resistance upon cell detachment in cancer cells [132]. Cancer cells exposed to shear stress or disturbed flow also activates TEAD via ROCK-LIMK-cofilin signaling, which promotes cancer cell motility and metastasis [133]. Furthermore, the activation of TEAD target gene, CTGF, mediates the metastatic colonization of breast cancer through leukemia inhibitory factor receptor (LIFR) suppression [134]. In colorectal cancer, RARγ promoted TEAD activation through the Hippo pathway, which induces EMT, invasion, and metastasis [135]. However, increased TEAD expression and its nuclear localization in colorectal cancer cells promote EMT and metastasis via a Hippo-independent mechanism [107]. Moreover, TEAD activation induces ROR1-HER3-mediated osteoclast differentiation and bone metastasis of cancer cells via the Hippo-YAP pathway [136]. MRTF-activated TEAD also promotes breast cancer cell metastasis to the lung [33]. In breast cancer and melanoma cells, SRC tyrosine kinase-induced cell-ECM adhesion activates TEAD to promote tumor growth and enhance metastasis [137]. In addition, TGFβ-induced TEAD transcriptional activity is required to promote metastatic phenotypes in breast cancer cells [72]. *ARHGAP29* is a TEAD target gene that increases cell invasion and metastasis by regulating actin dynamics in cancer cells [138]. TEADs are also involved in metastatic seeding via disseminated cancer cells. The spreading of circulating tumor cells induced TEAD activation via L1CAM-ILK-YAP signaling, which is critical for metastatic colonization [139]. Collectively, these studies suggest that TEAD activation enhances metastatic tumor formation and raises the possibility that TEAD inhibition can prevent the survival and outgrowth of disseminated tumor cells.

## 6. TEADs as Mediators of Cancer Genes 

Driver mutations in cancer-associated genes alter downstream signaling and transcription patterns, which are critical in tumor progression. Deregulation of TEAD transcriptional output have been demonstrated to mediate the pathology of critical oncogenes and tumor suppressor genes including NF2, BRAF, KRAS, MYC, PTEN, LKB1, and PKA (Figure 2a). This section will discuss the molecular mechanisms of TEAD regulation in the context of cancer gene-induced tumor development, drug resistance, and metastasis.

### 6.1. Hippo Pathway and TEADs

The Hippo pathway functions as a tumor suppressor pathway, whose activity is deregulated in many cancers [140]. However, mutations directly linked to alterations in the Hippo-YAP/TAZ pathway is uncommon. Inhibition of the core Hippo pathway components via point mutations and epigenetic alterations are found in subsets of human cancers including mutations in NF2 [141,142,143], MST1/2 [144,145], SAV1 [146], MOB1A/B [147], LATS1 [148,149,150,151,152], and LATS2 [88,153,154,155]. In addition, approximately 80% of uveal melanomas harbor activating mutations in GNAQ or GNA11, which are associated with the inhibition of the Hippo pathway and activation of TEAD [156,157]. Moreover, gain-of-function mutations were recently found in YAP and TAZ. Hyperactivating mutations in YAP were identified in melanoma and lung cancer patients [158,159,160]. Furthermore, chromosomal translocations that generated fusion proteins containing YAP (YAP-TFE3) and TAZ (WWTR1-CAMTA1) are oncogenic drivers in epithelioid hemangioendothelioma [161,162,163]. Similarly, in poromas and poro-carcinomas, fusion proteins containing YAP (YAP-MAML2, YAP-NUTM1) and TAZ (WWTR1-NUTM1) induced tumorigenesis via TEAD hyperactivation [164]. Several studies demonstrated that TEAD hyperactivation drives tumorigenesis driven via Hippo-YAP/TAZ pathway mutations, which suggests TEADs are therapeutic targets [141,143,157,158,162]. However, further investigations are required to link TEAD activity to the pathophysiology of each cancer gene within the Hippo pathway.

### 6.2. EGFR-RAS-RAF-MAPK Pathway and TEADs

TEADs are mediators of the EGFR-RAS-RAF-MAPK pathway, which is one of the most deregulated molecular pathways in human cancers. TEADs play important roles in tumor progression and drug resistance downstream of the hyperactivating mutations on EGFR, KRAS, or BRAF (Figure 2a). In NSCLC patients, TEAD activity correlates with the EGFR mutation status. EGFR mutant lung cancer tissues and cell lines show upregulated YAP expression, followed by increased TEAD activity [165]. Although EGFR acquires drug resistance to TKI via the T790M mutation, TEAD inhibition effectively reduces the viability of TKI-resistant lung adenocarcinoma cells [166,167]. TEAD also contributes to the immune escape of NSCLC cells by directly transcribing *PD-L1*, which, in turn, causes CD8^+^ T cell exhaustion [43,168]. Furthermore, TEAD increases EGFR expression by directly binding to the *EGFR* promotor, which induces tumorigenesis and drug resistance in esophageal cancer [39]. The KRAS oncogene, which is downstream of EGFR, is one of the most frequently mutated proteins in human carcinoma. Activating mutations in KRAS are particularly prominent in pancreatic ductal adenocarcinoma (PDAC). Unlike EGFR and BRAF, KRAS has, thus far, not been considered to be a viable therapeutic target, which renders downstream effectors critical in the treatment of PDAC. Activation of TEAD-induced target genes including *COX2* and *MMP7*, which fueled KRAS^G12D^ driven-PDAC progression both in vivo and in vitro, suggests Celebrex (COX2 inhibitor) and marimastat (a clinical MMP inhibitor) to be possible therapeutic agents for PDAC treatment [169]. Moreover, numerous TEAD target genes have been associated with unfavorable prognosis for PDAC [170]. TEAD2 was shown to act cooperatively with the E2F transcription factor to promote a cell-cycle gene expression program, which enabled the bypass of oncogenic KRAS addiction in PDAC and evoked KRAS-independent tumor relapse [171]. Approximately half of metastatic melanoma patients harbor the BRAF^V600^ mutation, with the most common being BRAF^V600E^ [172]. Although vemurafenib (PLX4032) and dabrafenib were developed to treat BRAF^V600^-mutant metastatic melanoma, a majority of patients ultimately became resistant [173]. Multiple studies have demonstrated that TEAD activity contributes toward BRAF inhibitor-resistance in melanoma cells. TEAD activity was increased in drug resistant-melanoma cells due to actin cytoskeletal remodeling that induced cancer stemness and invasion [174,175]. BRAF inhibitor-resistant cells can evade the immune system via TEAD activation. TEAD-mediated direct transcription of *PD-L1* was responsible for PD-1-dependent CD8^+^ T cell exhaustion, which allowed BRAF inhibitor-resistant cells to escape the immune responses [41,42]. Moreover, TEAD induced cytokines *IL-6* and *CSF1-3* in KRAS mutant PDAC cells to recruit myeloid-derived suppressor cells (MDSCs), which formed an immunosuppressive tumor microenvironment [176]. Collectively, the TEAD transcriptional output played critical functions in the pathogenesis of the EGFR-RAS-RAF-MAPK pathway and mutation-induced tumor progression.

### 6.3. LKB1-AMPK Signaling, Energy Stress, and TEADs

LKB1 (STK11) is a well-characterized tumor suppressor that governs diverse cellular processes, including cell growth, polarity, and metabolism. Approximately 15% to 30% of NSCLC patients harbor LKB1 inactivating mutations. An RNAi-based kinome screen identified LKB1-MARK signaling as a potent inhibitor of TEAD transcription activity by inactivating YAP, which suggests TEAD to be a therapeutic target in the treatment of LKB1-mutant human malignancies [177]. Another study showed that LKB1 was required by Dishevelled (DVL) to inhibit TEAD activity, which facilitated the co-activation of the Wnt/β-catenin and Hippo/YAP pathways [178]. Furthermore, the loss of LKB1 in lung adenocarcinoma cells activated TEAD, which directly transcribed *ZEB2* and repressed DNp63 to regulate cell fate and lineage conversion in lung cancer progression [130]. Similarly, the oncogenic protein Survivin, which is a TEAD target gene, promoted malignant progression of LKB1-deficient lung adenocarcinoma cells [179]. Downstream signaling of LKB1 led to the activation of AMPK, which is a key cellular energy sensor that functions as a tumor suppressor. Similar to LKB1, AMPK activation also inhibited TEAD activity. Upon energy stress, AMPK regulated TEAD by inhibiting YAP via both Hippo-dependent and Hippo-independent mechanisms, which resulted in impaired tumor growth [49,180,181]. Furthermore, the glucose transporter *GLUT3* is a direct target gene of TEAD. Studies have shown that the glycolytic enzyme PFK1 interacts with TEAD to increase glucose metabolism and tumor growth [49,73]. Further studies on the role of TEAD as a downstream mediator of LKB1-AMPK signaling and energy stress in growth control and cancer metabolism will provide important means of treating LKB1 mutant cancer cells. 

### 6.4. MYC and TEADs

The MYC oncogene is deregulated in >50% of human cancers and its hyperactivation is associated with a poor prognosis and unfavorable patient survival [182]. TEADs have emerged as transcription factors that induce *MYC* gene expression (Figure 2a). Transcriptome analysis of a Hippo-deficient gastric cancer model showed that TEAD activation directly upregulates *MYC* and its target genes, which, in turn, induced tumor progression [183]. Similarly, in oral squamous cell carcinoma (OSCC), TEAD induced *MYC* and *BCL-2* gene expression, which accelerated OSCC tumorigenesis [184]. In *Drosophila*, TEAD has also been shown to transcribe *MYC*, which induces tissue growth and increased organ size [44]. Furthermore, an in vitro cell competition assay shows that direct TEAD-induced *MYC* expression is required to become super-competitors, which indicates that TEAD and MYC cooperatively control cell proliferation and cell competition [185]. Despite several studies demonstrating MYC to be a TEAD target gene, MYC is also shown to act in parallel with or upstream of TEAD to modulate cellular function. MYC and TEAD coordinate the gene expression required for cell proliferation upon activation of mitogenic signals. TEADs were found to be constitutively bound to the promoters of the MYC target genes. The subsequent binding of MYC to these sites recruited YAP to bind TEAD, which induced MYC/TEAD target gene expression. This cooperativity between MYC and TEAD induced liver growth and tumorigenesis in mouse models [186]. Notably, the TEAD4-MYCN positive feedback loop was identified to drive high-risk neuroblastoma associated with MYCN amplification [45]. Apart from these reports, TEAD and MYC also showed an anti-correlation in breast cancer patients that enabled the stratification of breast cancer subtypes [187]. Another study also shows that MYC inhibits TEAD transcriptional activity in MYC-driven breast cancer by suppressing the binding between TEAD and YAP/TAZ via AMPK-induced phosphorylation [188]. Similarly, a transcriptional repressor TRPS1 and MYC are commonly co-amplified in breast cancers and TRPS1 inhibits YAP-dependent TEAD activity by direct binding [189]. Collectively, TEAD and MYC form a regulatory feedback mechanism that is important for proper growth control. Thus, further research on the TEAD-MYC circuitry may yield new insights into the treatment of diverse cancer subtypes.

## 7. Conclusions

In this review, we underscore the molecular mechanisms and functions of TEAD, which is involved in the multistep process of tumor progression, including tumor development, EMT, drug resistance, and metastasis (Figure 2b). Although oncogenic driver mutations in TEAD are yet to be identified, the TEAD transcriptional output was found to be responsible for human malignancies via the crosstalk between various oncogenic signaling pathways and cancer genes such as the Hippo pathway, EGFR-RAS-RAF-MEK pathway, LKB1, GNAQ/11, and MYC (Figure 2a). Despite the emerging role of TEADs as critical effectors in cancer biology, most research regarding TEAD regulation focuses on its major coactivators, YAP and TAZ. YAP/TAZ-independent regulatory mechanisms that govern TEAD activity, which include post-translation modifications, subcellular localizations, and upstream activating and inhibiting stimuli, have been hardly elucidated. Notably, recent studies revealed unprecedented regulatory mechanisms of TEAD such as nucleocytoplasmic shuttling and palmitoylation. These provide the first mechanistic insight in manipulating and targeting TEAD subcellular localization and post-translational modifications to be attractive therapeutic interventions to treat human malignancies. Although flufenamates, originally developed as a COX inhibitor, have been demonstrated to bind and inhibit TEAD functions, additional drugs that specifically bind TEAD with high selectivity and efficacy is yet to be developed. Since TEAD functions as the signaling nexus for critical pathways in tumor progression, future progress in elucidating regulatory mechanisms of TEAD and developing therapeutic interventions will initiate an exciting new arena for basic science as well as pharmaceutics.
